# Collectanea

**Published:** 1832-11

**Authors:** 


### COLLECTANEA.

**Floriferis ut apes in saltibus omnia libant,**
**Omnia nos, itidem, depascimur aurea dicta.**

### PATHOLOGY.

*Remarkable Cases indicative of the Transmission of Malignant Cholera by Human Intercourse.* By L. BRAULT, M.D. &c., Vendome.

A MAN named Poilpret (Pierre) lived at the extremity of one of the faubourgs of Vendome, which for five weeks had been afflicted with the cholera. On the morning of the 6th of July he left Vendome in charge of a public vehicle, and shortly before arriving at the village of Lisle, distant about two leagues north of Vendome, he was seized with the symptoms of cholera. He was taken to an inn in the village, where he went to bed, and died in twenty-four hours. Up to this time the commune of Lisle had been exempt from the disease.

The widow Etienne Chevallier acted as Poilpret's nurse, assisted him to the day of his death, remained beside the body, placed it in the shroud, and never left it for twenty-seven hours, when it was interred. This female was about fifty-five years old, in good health, of naturally gay and lively character, and perfectly fearless respecting the functions she had fulfilled. On the second day after the interment of Poilpret, namely, on the 10th of July, while engaged at field labour, about noon, she suddenly experienced colic pains, and urgent and severe diarrhœa soon set in. In four hours she took to bed, and in this condition she remained till the next day at noon, when vomiting and cramps were superadded to the previous symptoms. In the course of the day, blueness, coldness, absence of the pulse, rice-water evacuations, and all other symptoms of the true malignant cholera occurred. It is needless to pursue here the details of symptoms, or of the treatment under which she recovered by the 26th of July.

The widow Montafie, of the same village, lived next door to the little inn where Poilpret (the first case) died, and she occupied a room separated by a thin mud partition. She was aged about fifty, of bad health, addicted to intemperance. On the 14th July she was seized with the first symptoms of cholera, and she died on the second day.

The widow Ratier, also an inhabitant of Lisle, a middle-aged woman of weak frame, but in good health, made frequent visits to both the preceding patients, and she shrouded Montafie, who was buried on the morning of the 17th. Ratier continued well until the 22d, on the morning of which day she was seized with diarrhœa. The distinct symptoms of cholera soon after set in, and the disease ran its course, terminating in a recovery on the 2d of August.

The last case is perhaps more remarkable than the other. Theresa Montafie, aged twenty-three, a servant in a family of the name of Jury at Moncé, a quarter of a league from Lisle, repaired to her mother's to attend her during her illness on the 15th of July, and only quitted her on her death. She returned to Moncé affected with diarrhœa, which on the 22d July lapsed into decided cholera, from which she recovered after a tedious struggle.—*Journal Hebdomadaire, and Lancet.*

---

### PRACTICAL MEDICINE.

### *On the Use of Nitrate of Silver, as an Application in Burns and Scalds.* By J. C. Cox, Surgeon, F.L.S. &c.

I TOOK occasion, some time since, to call the attention of the profession to the use of the nitrate of silver in certain cutaneous affections, especially herpes zoster, for which I expressed the opinion that it was almost a specific; and the employment of that remedy in many severe cases subsequently has fully confirmed that opinion.

I now desire to recommend the use of the same remedy in burns and scalds, as one more fitted that any other with which I am acquainted to relieve the present suffering and obviate the future mischief. Where the burn is deep, and has destroyed the vitality of the cutis, of course no superficial application can do more than hasten the separation of the slough, and the spirits of turpentine is still probably the best application; but, where there is extensive superficial lesion, either producing intense redness and pain, or vesication, the nitrate of silver is certainly unrivalled. I have employed it both in very strong solution and also in the solid form; but, on the whole, I think the latter by far the best mode of application. The parts being moistened with cold water, the stick of caustic is passed over the whole surface, which may be afterwards covered with cotton wadding and bandaged, where that can be conveniently done. The application does not increase the suffering; all uneasiness quickly subsides, and in a day or two the only traces of injury will be the desquamation of the blackened cuticle. Where the process of vesication has commenced, it is immediately arrested; but if the vesications have been extensive, and the cuticle torn off, or adherent to the dress, there is no application which forms so effectual and complete a protection from the external atmosphere as the lunar caustic. In the latter case, it should be rolled tightly and rapidly over the surface. It appears to combine chemically with the albumen, and to form a covering most beneficial and efficient. Where extensive sloughs have formed, and there is a large granulating surface, the nitrate of silver, in the form of strong solution, applied with a camel's-hair brush, diminishes the irritability of the surface, prevents, to a great degree, the distressing itching, and hastens cicatrization. I would here state that much mischief is frequently done by the use of astringents, as the oxyde of zinc, &c., which causes the cicatrix to contract quickly, and thus to form seams and bridles, which produce frequently great deformity. A young lady, a patient of mine, in dressing for a party, set fire to her pelerine, and severely burned the neck, back, and shoulder. On the shoulder, a large patch was completely disorganized: to this I applied the spirits of turpentine. The other parts were either vesicated or severely scorched, extending over the back and neck: to these I applied the lunar caustic all over the surface, and wrapped the whole up in a cotton wadding. In half an hour she became quite easy, went to sleep, and omitted to take the anodyne which was prepared for her. The shoulder was the only part which required any protracted attention.

Master C——, while casting bullets, scalded his hand with the melted lead. He was in great pain, which was only temporarily relieved by cold. I applied the lunar canstic to the surface; the pain was immediately relieved, and the next day there was no soreness or inconvenience.

A boy's face was severely burnt by the explosion of gunpowder: the application of the nitrate of silver completely obviated the ill effects which would otherwise probably have arisen from the accident. The face certainly appeared grotesquely tattooed for a few days, by the application, but all traces were quickly removed.

The above are sufficient exemplifications of a mode of treatment which will, I am convinced, on trial, be found very beneficial.—*Med. Gazette.*

---

### MEDICAL JURISPRUDENCE.

*The Possibility of Voluntary Locomotion, to a considerable Distance, after the complete Division of the Carotid Artery and other large Vessels of the Throat.* From a Lecture on Medical Jurisprudence, by Professor Amos, at the London University.

On resuming his lectures after the Spring circuit, Mr. Amos stated to his class the following particulars concerning a late trial for murder, in which he had been professionally engaged.

At the Warwick assizes, a man of the name of John Danks was tried for the murder of Mary Green. The prisoner was a farmer's labourer of the better sort; he was married to a second wife, by whom he had no children. The deceased was a woman of loose character, having had six illegitimate children, and being far advanced in pregnancy with a seventh, which she had some time since sworn to the prisoner. He still, however, continued to occasionally cohabit with her. It appears that, on the evening on which the murder was committed (Saturday the 18th February last), the prisoner left his home. Soon after he made his appearance at Mary Green's, and, opening the door of her house, invited her out to talk with him. They were seen by the deceased's daughter conversing together for a few minutes, after which Green returned to her dwelling, but shortly afterwards went out again to meet the prisoner. The parties were then seen proceeding together to their usual haunt across the field, to the place in the plan marked Astley's Hovel.

The next material circumstance in the history of the murder is, that shortly after this a cry of distress was heard, which reached the house of a woman of the name of Hammond, and came from the direction of Astley's Hovel. Mrs. Hammond called up her neighbour John Harris, and besought him to find out what was the matter. He put on his hat, and as soon as he got out of his door he heard the cry repeated; he described it as a very dreadful cry; but after pursuing the direction in which it came for a little distance, he stopped to listen, and hearing no more of it, returned quietly to his house. Between eight and nine o'clock the next morning (Sunday) the body of the deceased was brought home, having been found in the road, about twenty-three yards from Astley's Hovel.

Immediately upon the alarm being given, the constable of Nun Eaton reached the spot where the body was lying in the road; he examined the hovel, and found that somebody had been cut there; he traced the blood from the hovel to a gate some yards from it on the road side, and over that gate to the spot where the deceased lay. The prisoner was speedily apprehended, and on his clothes were found some marks of blood; a button, with blood on it, had been picked up at the gate already mentioned, and this button was found to correspond with those of the prisoner's waistcoat. The knife with which the murder was committed was found in the adjoining wheat-field, and identified as belonging to the prisoner. At first the man attempted to exculpate himself by a weak alibi, and endeavoured to account for the blood on his clothes by saying that he had assisted in the killing of a pig; a story which was subsequently proved to be a falsehood. After the coroner's inquest, however, upon his being committed to jail, he made a declaration or confession to the constable to this effect: that he and Green had walked together to the hovel, and were there for about a quarter of an hour, "when," said he, "I up with my fist and struck her on the temples and knocked her down. I fell at the back of her, and held her down, and she hooted very loud;

I cut her once while I lay at the back of her, but it did not stop her hooting; and then I cut her again, and that stopped her hooting, and I was sure she was done for. I got up, left the hovel, and got over the gate, and came up the road to the top of the Abbey-end; and as I was coming along the road, about one hundred yards from the hovel, I thought I heard the footsteps of a man coming after, but upon turning round, I could see nobody. I then shut my knife, and threw it into the wheat-field, and made my way home, where I washed my hands, and went to bed." He added, that he fell at the woman's back in order to escape the flow of blood.

We shall now take notice of the condition in which the body was found. The constable deposed, that when he came to the spot he perceived the dead woman lying on her face, with one arm under her, and her head towards the Abbey street; it was impossible for any one, as the body then lay, and without moving it, to see that the throat was cut; her cap was not on her head, but off behind, and part of it stuck to the blood on her face. The body was taken home on a door, and kept there in the same position in which it was found, until it was seen by the surgeon. The constable's evidence went further, to the identification of the prisoner's footmarks made by him in running across a wheat-field adjoining the place where the body was found; he took the prisoner's boots and compared them with the footmarks, and found that they fitted exactly; and the identification was made still more satisfactory by comparing those marks with an impression taken in cement. In the hovel the constable deposed that he found a large quantity of blood upon the straw, and thence to the gate, he said, were also marks of blood upon the woodwork of the side of the hovel; also on the bars of the gate was a quantity of blood, and on the topmost bar were marks as though it had been pressed by a heavy hand.

The evidence of Mr. Richard Lloyd, the surgeon who was called in to see the body, was to the effect that he found, at the upper part of the throat, near the angle of the left jaw, a gaping incised wound, carried to the pharynx and commencement of the œsophagus, to the posterior part of, and obliquely down, the right side of the neck, to the fourth cervical vertebra; such a wound, he deposed, including a complete division of the trunk of the carotid artery, and all the principal branches of the external carotid and the jugulars, must of necessity be mortal, and occasion death immediately, or at least in a very short time; so short, as to render it highly improbable, but not impossible, that the woman could have gone the distance of twenty-three yards, besides getting over the gate, in that dreadful condition. The wound measured seven inches in length, and three in depth. There was also another incised wound two inches below the former, in front of the throat, but not deep enough to divide the trachea; with that wound alone, he said she might have been able to have reached even her home, which, indeed, he thought the cause of her leaving the hovel previous to her receiving the last mortal wound. Mr. Lloyd had assisted the constable in comparing the footsteps in the wheat-field with the plaster impressions. It should be mentioned, that this gentleman's evidence as to the nature of the wounds, and the almost utter impossibility of the larger one being inflicted in the hovel, was given before the prisoner's voluntary confession.

Such, then, were the principal facts connected with this murder. Danks was, of course, found guilty and executed. Of his guilt, no possible or reasonable doubt can be entertained; but still the circumstances of the case are of a very singular nature in a medico-legal point of view. Did the murderer really inflict the mortal wound in the hovel, or on the spot where the body was found? And if the former, is it not a very extraordinary fact that after such a wound, in which the main trunk of the carotid, its external branches, and the jugulars, were severed, the power of locomotion to so large an extent should still remain? Mr. Lloyd's opinion is obviously such as the majority of intelligent medical witnesses would have given, and such only as they would most probably feel themselves justified in giving; from all that experience and history have hitherto taught us, no other conclusion apparently could be arrived at. All the facts, however, and circumstances of the case being put together, and duly weighed, (and we are deeply indebted to Mr. James Williams Buchanan, the solicitor, of Nun-Eaton, for the great industry and ability with which he has collected the evidence,) we can come to no other conclusion than that the murderer's declaration was perfectly true; that both the wounds were inflicted in the hovel, and after all, that the miserable woman was able to get on her feet, to proceed to the gate, and over the gate, to the place where she fell at last from utter exhaustion.

The first wound, as Mr. Buchanan observes, was quite inconsiderable. It was situated within an inch of the clavicle, and very superficial; the integuments were merely cut through, and the cartilage of the trachea was scarcely grazed. From such a wound, the quantity of blood in the hovel could never, by any possibility, have flowed. On the other hand, it would seem that there had been from the second wound a most copious flow of blood, which was thrown off by the prominence of her belly (she being far advanced in pregnancy,) to the side of the hovel, as she passed along to the gate; then on the gate a similar transfer of blood was manifest, and there also were the marks of both her hands. The prisoner's hand was also marked on the gate, but at a different place. Mr. Buchanan further informs us, that he went himself over the ground, the Sunday after the trial, and it took him fifteen seconds to pass from the spot where the wound was inflicted, over the gate, to the place where the body was found; and in a subsequent experiment, in which he proceeded at a slower pace, it occupied him twenty seconds. The obstruction of the gate, he thinks, might be equivalent to eight or ten yards more.* It is a curious fact, observed by this gentleman, that there was scarcely any blood between the gate and where the body fell; but this he very reasonably accounts for, by her having guarded the wound with her cap, and closed it still further by holding down her head. There was, however, a large quantity of blood found to have flowed down between the breasts and lodged about the pubes; to such an extent, indeed, was it there accumulated, that the woman who stripped the body thought at first that there must have been an attempt made to take away the child; but this was shewn to be not the case, upon medical examination. There was also, it appears, a great absorption of blood by the under garments.

But in estimating the time that elapsed from the infliction of the wound till the unfortunate woman dropped, (that is, supposing the wound to have been inflicted in the hovel, and Dank's confession to be true,) other considerations must also enter into the account. The fifteen or twenty seconds calculated by Mr. Buchanan would seem to be considerably short of the actual time. We should begin to reckon from the moment in which the murderer "stopped her hooting," the trachea having most probably then been divided, together with the great blood-vessels, after which the murderer arose from his prostrate position, and having left her for dead, went his way. She in the mean time must be supposed to have lain tranquil, at least while Danks was in the hovel, and to have rallied after a temporary collapse, after which she had to regain her feet, and to set herself in motion in the direction along which she was traced. All this could not have been done in an instant; on the contrary, several seconds must have passed, how many it is quite impossible, with any degree of exactness, to conjecture, but probably not less than ten more, at the very least, must be allowed. Then it is not the mere time of crossing the gate that should be calculated, but the expenditure of strength and the loss of blood that must have occurred in accomplishing this object.

And many more difficulties doubtless will not fail to present themselves to the mind of whoever takes the circumstances of this curious case into consideration;

* It was an ordinary five-bar gate, three feet ten inches in height.

though probably, after all, the chief difficulty, as in the famous miracle of St. Denys, (without levity might it be alluded to,) shall be thought to have existed in those movements which had necessarily to be made in the first instance, and, as was observed in the case of the French miracle, perhaps we too shall be inclined to conclude that, "il n'y a que le premier pas qui coute." Once allow that the unfortunate woman was able, after her "hooting was stopped," and she had recovered her legs, to recover also her faculties, and to put herself in motion, and much of her remaining difficulties must vanish. At all events several seconds should be allowed for this period in estimating the whole time elapsed from the division of the great vessels until she sunk from exhaustion.

It may just be added, that, to the hour of his execution, the prisoner declared that he never touched the woman except in the hovel, where he left her for dead. He persisted, however, in affirming that he certainly heard a footstep when on the road, that of a third person, as he conceived, coming behind him; and in that conviction he died.—*Med. Gazette.*

---

### MISCELLANEOUS.

*Influence of Animal and Vegetable Matters upon Epidemic Cholera.* The Academy of Medicine in Paris recently discussed the question "whether emanations from animal and vegetable matters have an injurious influence in epidemic cholera?" The Academy decided the question in the negative. This opinion may to some appear paradoxical, as most authors are agreed in assigning to such emanations a very prejudicial effect on the animal economy, even in ordinary times: the facts, however, are all in favor of such a decision. Sewers of vast extent have been opened at Paris during the prevalence of the cholera; the works have been prosecuted with great activity, and the soil which has been removed has, of course, been exposed to the air in different parts of the city. The quarter of the Place Vendôme, though traversed by a vast sewer, has suffered very little from cholera; whilst the Faubourg St. Germain, where there are no works of the kind, has been visited by the disease in its most virulent form. In addition to this it may be remarked, that the victims to the cholera among the masons and excavators employed on the works were not more numerous than among any other class of workmen.

---

*Health of the Metropolis.* The whole population of London, by the last returns, is 1,474,069. The mortality amounts to about four and a half in every thousand, or somewhat less than one half per cent., a rate which is believed to be nearly the lowest which has prevailed in any large town that has been really visited by the cholera.—*Med. Gazette.*

